# A microfluidics-based wound-healing assay for studying the effects of shear stresses, wound widths, and chemicals on the wound-healing process

**DOI:** 10.1038/s41598-019-56753-9

**Published:** 2019-12-27

**Authors:** Jin-Young Lin, Kai-Yin Lo, Yung-Shin Sun

**Affiliations:** 1 Department of Physics, Fu-Jen Catholic University, New Taipei City, 24205 Taiwan; 2 Department of Agricultural Chemistry, National Taiwan University, Taipei, 10617 Taiwan

**Keywords:** High-throughput screening, Lab-on-a-chip, Collective cell migration

## Abstract

Collective cell migration plays important roles in various physiological processes. To investigate this collective cellular movement, various wound-healing assays have been developed. In these assays, a “wound” is created mechanically, chemically, optically, or electrically out of a cellular monolayer. Most of these assays are subject to drawbacks of run-to-run variations in wound size/shape and damages to cells/substrate. Moreover, in all these assays, cells are cultured in open, static (non-circulating) environments. In this study, we reported a microfluidics-based wound-healing assay by using the trypsin flow-focusing technique. Fibroblasts were first cultured inside this chip to a cellular monolayer. Then three parallel fluidic flows (containing normal medium and trypsin solution) were introduced into the channels, and cells exposed to protease trypsin were enzymatically detached from the surface. Wounds of three different widths were generated, and subsequent wound-healing processes were observed. This assay is capable of creating three or more wounds of different widths for investigating the effects of various physical and chemical stimuli on wound-healing speeds. The effects of shear stresses, wound widths, and β-lapachone (a wound healing-promoting chemical) on wound-healing speeds were studied. It was found that the wound-healing speed (total area healed per unit time) increased with increasing shear stress and wound width, but under a shear stress of 0.174 mPa the linear healing speed (percent area healed per unit time) was independent of the wound width. Also, the addition of β-lapachone up to 0.5 μM did not accelerate wound healing. This microfluidics-based assay can definitely help in understanding the mechanisms of the wound-healing process and developing new wound-healing therapies.

## Introduction

Collective cell migration plays important roles in various physiological processes such as embryonic development, tissue repair, angiogenesis, and wound healing^1,2^. Recently, researchers found that collective cell migration is highly involved in the invasion and spread of malignant cells^3^. When migrating collectively, cells often form the so-called self-assembled monolayers where they are attached to each other in mechanical and biochemical ways. This complicated phenomenon is observed to occur in cellular proliferation, cell-cell communication, and cell-micro-environment interaction. To investigate how cells migrate collectively, a number of different *in vitro* techniques have been developed^4–8^. For example, wound-healing assays are routinely used because they are convenient and easy-to-use. In these assays, cellular shape and collective movement can be followed in real-time for quantitative analysis of morphology and migration speed.

In a typical wound-healing experiment, it is necessary to create a “wound” out of a monolayer of cells. Then surrounding cells grow and migrate to cover this empty space^9^. For example, in the scratch wound-healing assay, a tip or needle is used to create a cell-free region^10,11^. This assay has been commercialized for investigating collective cell migration (the CellPlayer Migration Assay by Essen BioScience). Advantages of this assay include easy and quick procedures, applicability to any substrates, and easy records of cellular morphology and migration^12^. However, it does have some limitations^13^. First, in creating the cell-free region, the number of removed cells and the shape of the region can be different from experiment to experiment. Therefore, it is difficult to make a comparison in between. Second, in creating a wound, cells may be damaged mechanically, leading to the release of certain cellular chemicals into the micro-environment. The degree to which cells are damaged cannot be easily controlled, so the complexity in analyzing cell migration will highly increase. Thirdly, when an extracellular matrix (ECM) is coated on the surface for better cell attachment and proliferation, a mechanical scratch may cause partial loss or damage to the ECM. In contrast, the barrier wound-healing assay is more suitable for cell migration studies^13,14^. It provides a controllable wound shape/size and maintains surface integrity by using a barrier to keep cells away from the wound. This assay has been reported to give similar wound-healing responses compared to the scratch-based one^15–17^. In conducting the barrier wound-healing assay, a barrier is put onto the substrate prior to seeding cells to grow into a confluent monolayer, and then the barrier is removed to allow cells to migrate and recover the gap. Cell Biolabs (San Diego, CA USA) has commercialized the barrier assay named the CytoSelect™ Wound Healing Assay. Other wound-healing assays such as laser photoablation and electrical wound-healing assays have also been developed as substitutes^13,18–20^. Recently, our group developed an alternative wound-healing assay based on ultraviolet (UV) light ablation^21^. As reported, this UV wound-healing assay resulted in similar wound closure responses compared to the scratch assay, and it provides unique advantages such as fast, easy procedure and high throughput^21^.

In most of above-mentioned wound-healing assays, cells are cultured in open, static (non-circulating) systems. Experimental results found in these *in vitro* assays could be different from those occurring *in vivo*^22^. Therefore, microfluidic devices provide a more suitable platform for both culturing cells in an *in vivo*-like micro-environment and applying external stimuli in a precise and controllable manner. The first microfluidics-based wound-healing assay was reported by Nie *et al*. in 2007^23^. Trypsin solutions in microfluidic channels form laminar flows to detach a portion of cellular monolayer, creating wounds similar to those occur *in vivo*. A migration distance of around 600 μm was observed after treating cells with epidermal growth factor-contained culture medium for 24 hr. Other similar microfluidics-based assays were reported to study wound-healing processes of various cell types including vascular smooth muscle cells^24^, endothelial cells^25^, and alveolar epithelial-like cells (A549)^26,27^. However, all these assays were limited to creating only one wound in one experiment, and the external stimuli to be applied were limited to chemicals. To increase the experimental throughput as well as to apply various physical/chemical stimuli, here we designed and developed a microfluidic chip serving as a novel, alternative wound-healing assay. By using the trypsin flow-focusing technique, this assay is capable of creating three or more wounds of different widths. The effects of shear stresses, wound widths, and β-lapachone on wound-healing speeds were studied. Although some scratch-based, barrier-based, and laser photoablation-based wound-healing assays have been reported to be high throughput^19,28^, the present microfluidic device can perform the assay in a non-contacting, fluid-circulating, and increased-throughput (compared with other microfluidics-based wound-healing assays) manner. This will definitely help in understanding the *in vivo* mechanisms of the wound-healing process.

## Results and Discussions

### Simulation and calculation of laminar flow and shear stress

**Figure 1:**
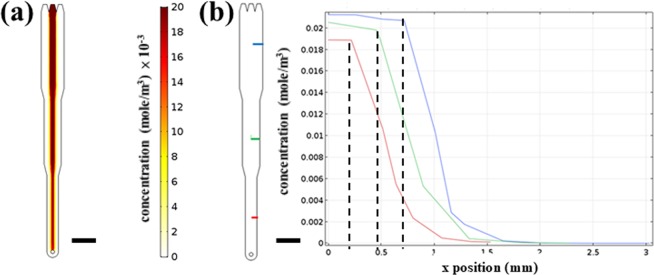
(**a**) 3D numerical simulation of trypsin concentration inside the microfluidic chip. The concentrations in the central and side inlets were 0.0214 mole/m^3^ and 0 mole/m^3^, respectively. Scale bar = 6 mm. (**b**) 1D concentration profiles along different widths of the microfluidic chip. Three colors represent *x* positions along three different widths, starting from the middle to the right, as shown in the left (blue: the 6-mm width; green: the 4.5-mm width; red: the 3-mm width). Scale bar = 6 mm. The dash lines indicate the regions where the concentrations of trypsin were close to the initial value of 0.0214 mole/m^3^.

Figure 1a shows the three-dimensional (3D) numerical simulation of trypsin concentration inside the microfluidic chip, where the concentrations in the central and side inlets were set to be 0.0214 mole/m^3^ and 0 mole/m^3^, respectively. As clearly seen, laminar flows were formed and flow-focusing was achieved inside the chip. Although the concentrations decreased from the upstream to the downstream, being average 0.0212 mole/m^3^, 0.0205 mole/m^3^, and 0.018 mole/m^3^ in the central flows of Area 1, Area 2, and Area 3, respectively, the values were close to the initial 0.0214 mole/m^3^. The one-dimensional (1D) concentration profiles along the widths of the microfluidic chip are shown in Fig. 1b. A stable trypsin concentration was formed in the center flow, which was used to detach cells and create the wound. As indicated by the dash lines, the regions where the trypsin concentration was constant had widths of around 1.42 mm, 0.91 mm, and 0.46 mm in Area 1, Area 2, and Area 3, respectively (double the lengths between zero and the dash lines). These values corresponded to 24%, 20%, and 15% of the channel widths (width = 6 mm, 4.5 mm, and 3 mm in Area 1, Area 2, and Area 3, respectively) and could be compared with the widths of the actual wounds.

The shear stress inside the microfluidic chip was also simulated (data not shown). The simulated together with calculated values under different flow rates are listed in Table 1. In the treatment stage, the flow rate was set to be 1200 μL/min (800 μL/min in the side inlet and 400 μL/min in the central inlet), giving simulated shear stresses of around 158 mPa, 213 mPa, and 328 mPa in Area 1, Area 2, and Area 3, respectively. These extreme stresses were used to strip off cells and lasted for 10 min. In the healing stage, the flow rate was varied from 40 μL/hr to 400 μL/hr to investigate the effects of shear stresses on the wound-healing process. These rates corresponded to simulated shear stresses of 0.088 mPa (Area 1 under 40 μL/hr) to 1.83 mPa (Area 3 under 400 μL/hr). As indicated in the table, the errors between simulated and calculated values were in general less than 5%.

**Table 1:** Simulation and calculation of the shear stresses inside the microfluidic chip.

	Width (mm)	Shear stress (mPa)	Treatment stage flow rate (µL/min)	Healing stage flow rate (µL/hr)			
			1200	40	80	200	400
Area1	6	Simulation	158	0.088	0.176	0.441	0.882
		Calculation	156	0.0872	0.174	0.436	0.872
		Error (%)	1.28	0.92	1.15	1.15	1.15
Area2	4.5	Simulation	213	0.119	0.238	0.594	1.19
		Calculation	208	0.116	0.232	0.581	1.16
		Error (%)	2.4	2.59	2.59	2.24	2.59
Area3	3	Simulation	328	0.183	0.366	0.914	1.83
		Calculation	312	0.174	0.348	0.872	1.74
		Error (%)	5.13	5.17	5.17	4.82	5.17

### Flow focusing to create wounds

**Figure 2:**
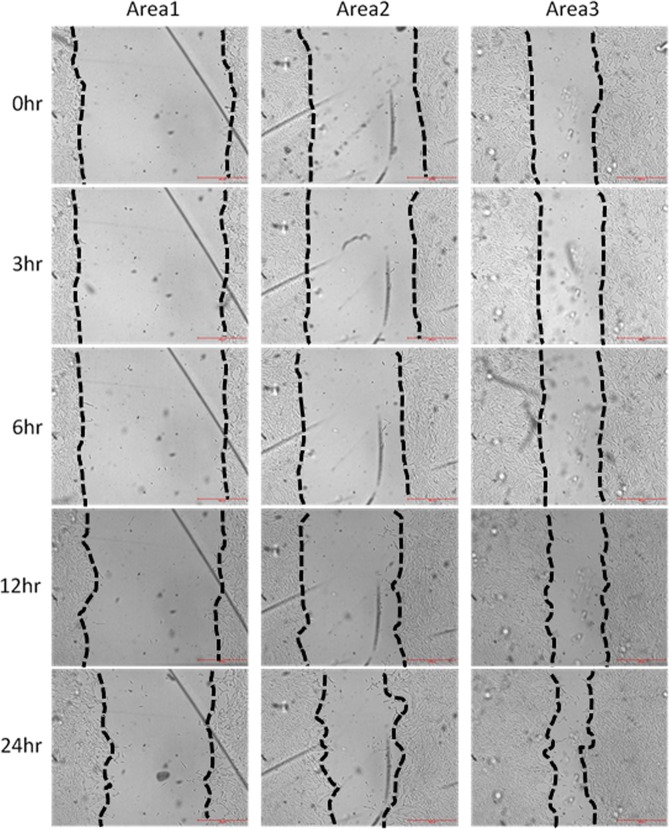
Pictures of the wound-healing processes under a flow rate of 200 μL/hr and a β-lapachone concentration of 0.5 μM. Scale bar = 500 μm.

Figure 2 shows representative pictures of the wound-healing processes under a flow rate of 200 μL/hr and a β-lapachone concentration of 0.5 μM in the healing stage. The wound widths in Area 1, Area 2, and Area 3 were around 1.45 mm, 1.05 mm, and 0.59 mm, respectively. These values corresponded to 102%, 115%, and 128% of the widths where the trypsin concentration was constant in the simulation (see Fig. 1b). By increasing the number of segments having different widths in the microfluidic chip, more wounds of different sizes could be created to enhance the experimental throughput. After creating the wounds, time-lapse images were taken at 0 hr, 3 hr, 6 hr, 12 hr, and 24 hr to calculate the wound-healing speeds in the unit of μm^2^/min under different conditions.

### Effects of shear stress on wound-healing process

Fluidic shear stresses were reported to affect cells in various aspects including attachment, morphology, alignment, living cycle, and damage^29–31^. In the molecular level, these stresses could alter gene expression and signaling cascades in cells such as retinal microvascular endothelial cells^32^, Schlemm’s canal cells^33^, and corneal endothelial cells^34^. Especially for endothelial cells, since they are *in vivo* exposed to continual flows, wound closure must occur under fluidic shear stresses. Wounds occurring in the endothelium are critical to a variety of cardiovascular disorders such as ischemic injury, vein bypass graft failure, coarctation the aorta, and mechanical trauma^35^.

**Figure 3:**
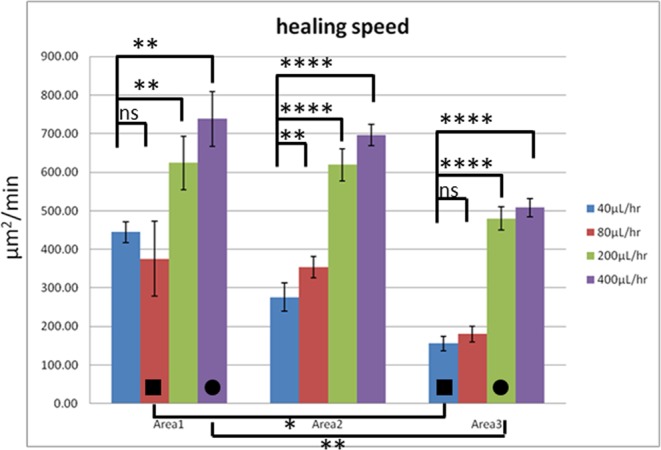
Wound-healing speeds under different wound widths and flow rates. The number of experiments *N* = 9, and the error bars represent the standard errors of the mean (SEM) (see Data analysis in Methods). Statistical analysis was performed. ns: no statistically significant difference (p > 0.05); *p < 0.05; **p < 0.01; ***p < 0.001; ****p < 0.0001.

The wound-healing speeds after 24 hr under different healing-stage flow rates are shown in Fig. 3. By changing the flow rate from 40 μL/hr to 400 μL/hr, the shear stresses varied from 0.087 mPa, 0.116 mPa, and 0.174 mPa to 0.872 mPa, 1.16 mPa, and 1.74 mPa in Area 1 (width = 6 mm), Area 2 (width = 4.5 mm), and Area 3 (width = 3 mm), respectively (see Table 1). As indicated in this figure, in Area 1, a 1.7-fold increase in the speed was noticed as the shear stress became ten times larger. With the same enhancement in the fluidic shear stress, the healing speeds exhibited 2.5- and 3.3-fold increases in Area 2 and Area 3, respectively. By plotting the wound-healing speeds against shear stresses in three different widths (see Fig. S1), the ranges of shear stresses in these three widths are very close. In the overlapping range (from 0.174 mPa to 0.872 mPa, marked between two dash lines), the healing speed in general increased with an enhanced shear stress.

Conventionally, the wound-healing assays are conducted under static conditions (in petri dishes for example) without the mechanical cues of shear stresses. It was reported that the shear stress accelerated the wound-healing process in human umbilical vein endothelial cells (HUVECs) and human coronary artery endothelial cells (HCAECs)^36^. For example, HUVEC wounds exposed to shear stresses of 3 dyn/cm^2^, 12 dyn/cm^2^, and 20 dyn/cm^2^ closed to 21%, 39%, and 50% of original wounding areas after 6 hr, respectively, compared to only 59% in the static condition^36^. Gojova *et al*. found that bovine aortic endothelial cell wound closure under flow was critically sensitive to the shear stress level to which the cells were exposed ^37^. After 12 hr, low levels of shear stress (3 dyn/cm^2^) led to dramatically retarded wound closure rates relative to high shear stress levels (19 dyn/cm^2^)^37^. However, other studies revealed totally contrary results using human corneal epithelial cells (HCECs) as the model. It was reported that after 24 hr, the wound-healing rate at 12 dyne/cm^2^ decreased significantly compared with the static control^38^. A possible reason was that fluidic shear stress on the HCECs affected transforming growth factor-β signaling, which was associated with delayed wound healing^38^. Another study showed that the wound-healing process was impaired in both low (4 dyne/cm^2^) and high (8 dyne/cm^2^) shear stresses after 48 hr, compared to the no-shear condition^39^. HCECs exposed to shear stresses showed cytoskeletal rearrangement with more prominent, organized and elongated filamentous actin^39^, and this phenomenon has been shown to be related to the migration and proliferation of epithelial cells^40,41^. For fibroblasts, four hours of 20 dyn/cm^2^ shear stress significantly enhanced sub-confluent (30 ~ 50% confluence) fibroblast migration while it suppressed full-confluent fibroblast migratory activity^42^. However, four hours of 1 dyn/cm^2^ shear stress did not significantly alter either sub- or full-confluence fibroblast migration levels^42^. It was also found that fibroblasts exposed to fluid shear stress structurally rearranged pre-coated surface fibronectin^43^. Summarizing these results, we suggest that the effects of fluidic shear stress on the wound-healing process could be cell type-dependent, and the underlying mechanisms remain to be studied.

### Effects of wound width on wound-healing process

The main advantage of the present microfluidic chip is that wounds of different widths could be created simultaneously to follow their healing processes. As indicated in the two columns with square marks in Fig. 3, under a shear stress of 0.174 mPa (see Table 1), the wound-healing speed decreased from 375.5 μm^2^/min to 155.8 μm^2^/min as the wound width was reduced from 1.45 mm to 0.59 mm (observation values). Similarly, when the shear stress was increased to 0.872 mPa (see Table 1), a decrease in the healing speed from 738.6 μm^2^/min to 479.7 μm^2^/min was observed with a reduced wound width (see the two columns with circle marks in Fig. 3). It seems that cells tended to migrate faster as the cell-free area was larger. However, in clinical trials investigating the influence of wound geometry on the measurement of wound healing rates, it was proposed that not the total area healed per unit time but the linear growth of wound edge per unit time should be calculated and compared^44^. This means that the percent area healed per unit time should be determined, and in the present study the initial widths of the wounds should be normalized. From this point of view, under a shear stress of 0.174 mPa, the normalized wound-healing speeds for two different widths were both close to 0.26 μm/min. But under a higher shear stress of 0.872 mPa, the normalized wound-healing speed for the 0.59-mm-width wound was about 1.6 times larger than that for the 1.45-mm-width one. Under a lower shear stress (0.174 mPa) the percent area healed per unit time was independent of the size of the wound. But at a higher shear stress (0.872 mPa) the percent area healed per unit time increased with a decreasing wound width. This trend is generally true as shown in Fig. S2 where the normalized wound-healing speeds are plotted against shear stresses in three different widths. In the overlapping range (from 0.174 mPa to 0.872 mPa), the normalized healing speed was independent of the size of the wound under a low shear stress (all close to 0.25 ~ 0.3 μm/min), but this speed increased with a decreasing wound width under a higher shear stress.

To the best of our knowledge, the effects of wound widths on the wound-healing process have only been rarely studied due to the difficulty in creating wounds of precise sizes and shapes. In clinical trials involving 39 patients with venous stasis ulcers, it was found that the linear healing per day was not affected by any geometric variable, including area, perimeter, length, width, and ratio of width to length^44^. Without presenting quantitative data, another study concluded that the wound-healing response of bovine corneal endothelial cells depended only on the presence of extracellular matrix (ECM) but not on the size and geometry of the wounds^45^. Using NIH/3T3 fibroblasts as the model, the effects of initial geometry on the wound-healing process were investigated^46^. As reported, the linear healing rates were similar in three different wound geometries including square, circle, and triangle^46^. All these findings, both *in vivo* and *in vitro*, were similar to what we have observed in the low shear stress case. Experimental results similar to those observed in the high shear stress case were not found; therefore, detailed investigations involving cell-cell communication and signaling under different shear stresses and wound sizes are required.

### Effects of β-lapachone on wound-healing process

**Figure 4:**
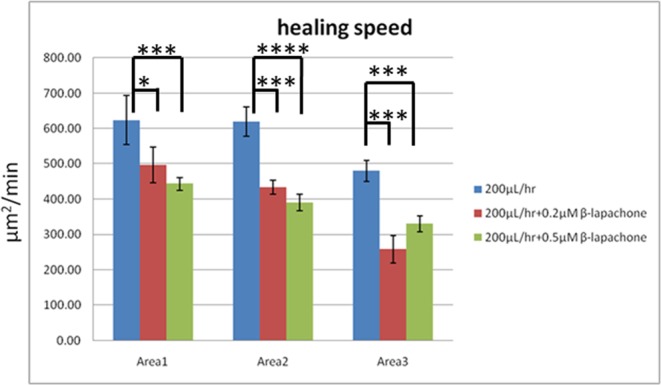
Wound-healing speeds under different wound widths and β-lapachone concentrations. The number of experiments *N* = 9, and the error bars represent the standard errors of the mean (SEM) (see Data analysis in Methods). Statistical analysis was performed. ns: no statistically significant difference (p > 0.05); *p < 0.05; **p < 0.01; ***p < 0.001; ****p < 0.0001.

β-lapachone, a derivative of naturally occurring lapachol, was shown to exhibit various pharmacological properties including anti-bacterial, anti-inflammatory, anti-angiogenic, anti-metastatic, and anti-invasive effects^47,48^. For studying its effects on the wound-healing speed in a dose-dependent manner, it was flowed into the microfluidic chip at a healing-stage flow rate of 200 μL/hr. Surprisingly, as shown in Fig. 4, β-lapachone in general decreased the healing speed. In Area 1, the speeds were 623.8 μm^2^/min, 496.2 μm^2^/min, and 443.4 μm^2^/min under β-lapachone concentrations of 0 μM, 0.2 μM, and 0.5 μM, respectively. In Area 2, the speed decreased from 619.3 μm^2^/min without β-lapachone to 433.8 μm^2^/min and 390.5 μm^2^/min when concentrations of 0.2 μM and 0.5 μM were added, respectively. In Area 3, the speed decreased as 0.2 μM β-lapachone was added, but the value increased when the concentration was 0.5 μM.

β-lapachone was shown to have wound healing-promoting activities both *in vivo* and *in vitro*^49,50^. Application of ointment with β-lapachone to punched wounds in normal and diabetic mice showed accelerated wound-healing processes^50^. Also, a low dose of β-lapachone (up to 1 μM) enhanced the proliferation in several cells, facilitated the migration of mouse 3T3 fibroblasts, and accelerated scratch-wound healing *in vitro*^50^. By using a microfluidic electrical-stimulated wound-healing chip, it was reported that β-lapachone alone (up to 2 μM) or low concentrations of β-lapachone (up to 0.5 μM) combined with electric fields (EFs) increased the wound-healing rate of NIH/3T3 cells, but overdose of β-lapachone (over 1 μM) in the presence of EFs decreased the rate instead probably due to excess ROS production^51^. From above-mentioned findings, the effects of β-lapachone on the wound-healing process were uncertain, which could depend on experimental conditions such as its concentration, the type of cells, the type of the assay (ex: scratch, barrier…), the micro-environment (ex: static or circulating), and the application of other stimuli (ex: EF…). Again, further studies are necessary.

### Limitations of the present microfluidic chip

**Figure 5:**
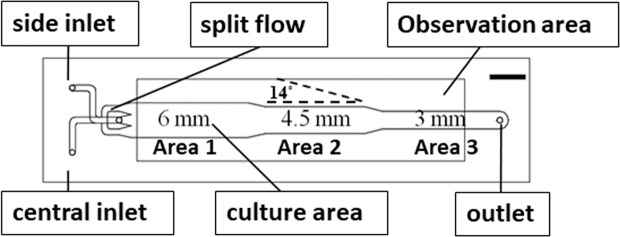
Design of the microfluidic chip. Scale bar = 6 mm.

Currently wounds of three different widths were created, and this is not very high throughput. But it did increase compared with other microfluidics-based wound-healing assays (where only one wound width was created). The throughput of the present chip can be further increased by increasing the number of different widths in the microfluidic channel (see Fig. 5, currently 3, 4.5, and 6 mm). The inability to vary wound width and shear stress independently is another limitation of this device. To study the dependence of wound-healing process on the shear stress or on the wound width, additional experiments are required. Finally, the fact that one wound is downstream from another means that the cells at the beginning of the chip could be releasing molecules that affect the wound healing downstream. This a general issue in fluid-circulating, microfluidic-based wound-healing assays.

## Conclusion

The wound-healing assay is a useful method for investigating collective cell migration under various chemical and physical stimuli. Traditional scratch- and barrier-based assays have drawbacks of unprecise wound sizes/shapes, damages to cells/substrate, and experiment-to-experiment variations. In this paper, we reported a microfluidics-based wound-healing assay to study the effects of shear stresses, wound widths, and β-lapachone on the wound-healing process. By using the trypsin flow-focusing technique, wounds of different widths were created. Experimental results indicated that the wound-healing speed increased with increasing shear stress and wound width, but under a 0.172 mPa shear stress the linear healing speeds were similar for two different widths. The addition of β-lapachone up to 0.5 μM did not accelerate wound healing. The present platform can serve as an alternative assay for further investigating the mechanisms of the wound-healing process in a non-contacting, fluid-circulating, and increased-throughput manner.

## Methods

### Chip design and fabrication

**Figure 6:**
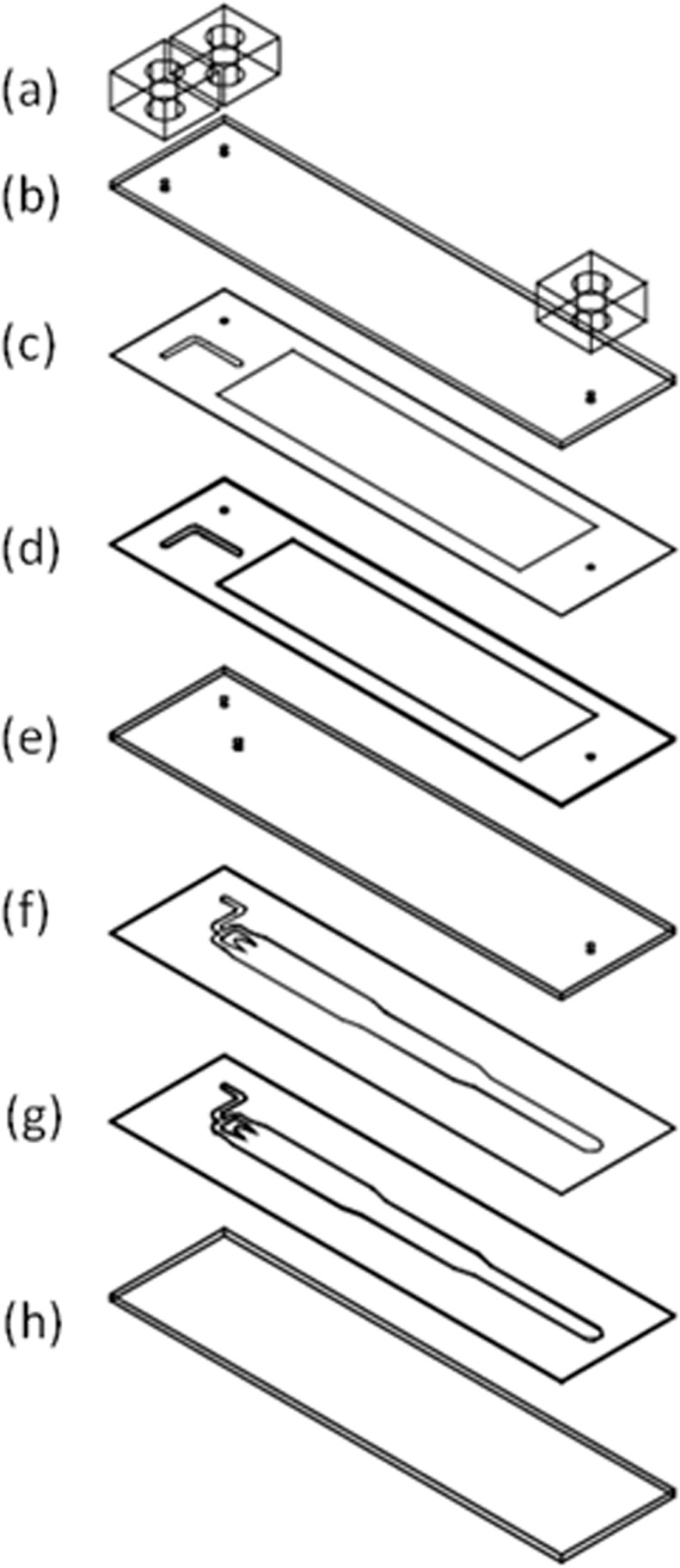
Layer-by-layer structure of the microfluidic chip. (**a**): adaptors; (**b**), (**e**), and (**h**): 1-mm PMMA substrate; (**c**) and (**f**): 60-μm double-side tape; (**d**) and (**g**): 260-μm double-side tape.

The design and fabrication of the microfluidic chip is shown in Fig. 5. It consisted of a central inlet for trypsin flow, a side inlet for medium flow, and an outlet. The cell culture area had three different widths, 6 mm (Area 1), 4.5 mm (Area 2), and 3 (Area 3) mm, for creating wounds of different sizes. The shrinking angle between two widths was set to be around 14° to prevent air bubble formation near the corners. The pattern was drawn in AutoCAD (Autodesk) and then loaded into a CO_2_ laser scriber (MS640D, Ming-Cheng Technics Corp.) to ablate desired patterns on polymethylmethacrylate (PMMA) substrates and double-sided tapes (8018, 3 M). As shown in Fig. 6, seven layers of PMMA sheets (thickness = 1 mm) and double-sided tapes (thickness = 260 μm and 60 μm) were bound together to form the integrated chip. The top PMMA layer had three small holes/adaptors serving as flow inlets and outlets. The bottom two double-sided tapes ((f) and (g) in Fig. 6) provided the fluidic channel for trypsin flow-focusing and cell culture, having a total thickness of 320 μm. Cytotoxicity of PMMA substrates and double-sided tapes on cells was examined, and no significant change in cell viability was observed^52^.

### Simulation and calculation of laminar flow and shear stress

The numerical simulation of chemical concentration and shear stress inside the microfluidic chip was performed using the commercial software COMSOL Multiphysics (ver. 5.2a, COMSOL). The “Laminar Flow” and “Transport of Dilute Species” modules were used with the following parameters and settings: the boundary condition for inlets is “velocity”: normal inflow velocity; the boundary condition for outlet is “pressure”: 101,300 Pa with normal flow and suppress backflow; diffusion coefficient = 2.1 × 10^−10^ m^2^/s for trypsin in culture medium at room temperature; central inlet flow rate = 400 μL/min; side inlet flow rate = 800 μL/min; concentration = 0.0214 mole/m^3^ for trypsin solution in central inlet; concentration = 0 mole/m^3^ for medium in side inlet. For calculation in Table 1, the shear stress (τ) within the cell culture area is related to the volume flow rate (*Q*), fluidic viscosity (η), and dimensions of the channel (height *h* and width *w*) as ${\rm{\tau }}=6\frac{\eta Q}{{h}^{2}w}$^53^.

### Cell preparation

The procedures were detailed in a previous study^54^. The mouse embryonic fibroblast cell line NIH/3T3 was purchased from the Bioresource Collection and Research Center (BCRC), Taiwan. A complete cell culture medium composed of Dulbecco’s modified Eagle medium (DMEM, Gibco) and 10% calf serum (CS, Invitrogen) was used for cell culture. The cells were incubated in tissue culture polystyrene flasks (Corning) in 5% CO_2_ at 37 °C until 90% confluence before seeding into the microfluidic chip.

### Experimental system and procedure

**Figure 7:**
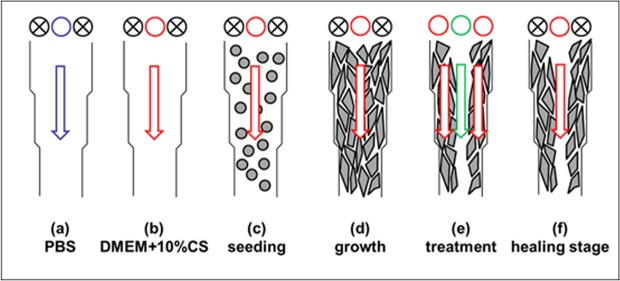
Experimental procedure for creating wounds. Blue: washing buffer (PBS); Red: cell culture medium (DMEM + 10% CS); Green: 0.05% trypsin; Gray: cell.

The procedures were detailed in a previous study^55^. The microfluidic chip was assembled inside the laminar flow hood and then UV-sterilized for 30 min. The central inlet was connected to a syringe driven by a syringe pump (NE-300, New Era). The experimental procedure is illustrated in Fig. 7. 1× phosphate-buffered saline (PBS) buffer was flowed into the chip (see Fig. 7a) and kept within the channel under 5% CO_2_ at 37 °C for 2 hr. The syringe was pushed gently by hands to remove bubbles, and PBS buffer was replaced with cell culture medium (see Fig. 7b). 3 × 10^6^ cells suspended in 1 mL culture medium was loaded into the culture area by injecting the cell solution from this inlet (Fig. 7c). The microfluidic chip with seeded cells was first incubated for 2.5 hr. It was then mounted on top of a transparent indium tin oxide (ITO) glass (Part No. 300739, Merck) for maintaining the temperature at 37 ± 0.5 °C^55^. Fresh culture medium was continuously pumped into the chip at a flow rate of 40 μL/hr until a cell monolayer was formed (Fig. 7d).

After a cellular monolayer was formed, the syringe was disconnected from the central inlet. Two new syringes, one filled with culture medium and the other filled with 0.0214 mole/m^3^ (0.05% w/w) of trypsin (Sigma) solution, were connected to side and central inlets, respectively. Flow rates of 800 μL/min and 400 μL/min were set in side and central inlets, respectively. All liquids were continuously flowed into the chip for 10 min (Fig. 7e), and then the trypsin solution was replaced with culture medium for washing off dead cells. To observe the wound-healing process, the central inlet was continuously flowed in culture medium (Fig. 7f) with or without adding the chemical β-lapachone (0.2 μM or 0.5 μM in culture medium, Sigma). Different flow rates were set to obtain desired shear stresses. Time-lapse images were taken at 0 hr, 3 hr, 6 hr, 12 hr, and 24 hr after the wound was created.

### Data analysis

**Figure 8:**
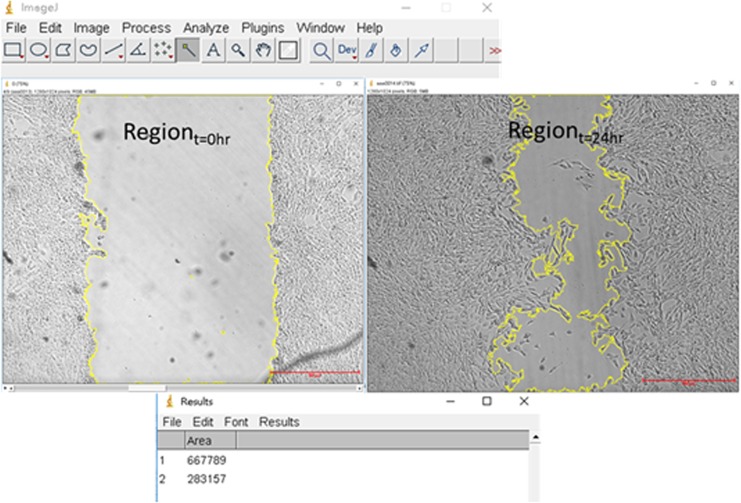
ImageJ was used to draw the boundaries and calculated the area of the wound. Scale bar = 500 μm.

The wound-healing process was recorded using a bright-field inverted microscope (ESPA, Hsinchu, Taiwan). Time-lapse images were taken at 0 hr, 3 hr, 6 hr, 12 hr, and 24 hr after the wound was created. Under a certain condition, three images were recorded for each area (at different locations) at one time point. They were further analyzed using ImageJ, which is a free Java-based software developed by the National Institutes of Health (NIH, Bethesda, MD). As shown in Fig. 8, this software was used to draw the boundaries of the wound at different time points. The area enclosed by these boundaries could be calculated. And the wound-healing speed is calculated as $\frac{({R}_{i}-{R}_{f})}{T},$where *R*_*i*_ and *R*_*f*_ are the initial an final areas of the wound, respectively, and *T* is the time after the wound was created. For each condition, three independent runs were performed. Therefore, there were total nine (three images times three runs) data to be analyzed to get the standard errors of the mean (SEM) as the error bars in Figs. 3 and 4.

## Supplementary information

Supplementary Figure.

## References

[1] Friedl P, Gilmour D (2009). Collective cell migration in morphogenesis, regeneration and cancer. Nature reviews. Molecular cell biology.

[2] Rorth P (2009). Collective cell migration. Annual review of cell and developmental biology.

[3] Deisboeck TS, Couzin ID (2009). Collective behavior in cancer cell populations. Bioessays.

[4] Chen HC (2005). Boyden chamber assay. Methods Mol Biol.

[5] Li YH, Zhu C (1999). A modified Boyden chamber assay for tumor cell transendothelial migration *in vitro*. Clin Exp Metastasis.

[6] Somersalo K, Salo OP, Bjorksten F, Mustakallio KK (1990). A simplified Boyden chamber assay for neutrophil chemotaxis based on quantitation of myeloperoxidase. Anal Biochem.

[7] Jungi TW (1975). Assay of chemotaxis by a reversible Boyden chamber eliminating cell detachment. Int Arch Allergy Appl Immunol.

[8] Hasan Jurjees, Shnyder S.D., Bibby M., Double J.A., Bicknel R., Jayson G.C. (2004). Quantitative Angiogenesis Assays in vivo – A Review. Angiogenesis.

[9] Todaro GJ, Lazar GK, Green H (1965). The initiation of cell division in a contact-inhibited mammalian cell line. J Cell Physiol.

[10] Friedl P, Hegerfeldt Y, Tusch M (2004). Collective cell migration in morphogenesis and cancer. Int J Dev Biol.

[11] Yarrow JC, Perlman ZE, Westwood NJ, Mitchison TJ (2004). A high-throughput cell migration assay using scratch wound healing, a comparison of image-based readout methods. BMC Biotechnol.

[12] Cory G (2011). Scratch-wound assay. Methods Mol Biol.

[13] Riahi Reza, Yang Yongliang, Zhang Donna D., Wong Pak Kin (2012). Advances in Wound-Healing Assays for Probing Collective Cell Migration. Journal of Laboratory Automation.

[14] Kroening S, Goppelt-Struebe M (2010). Analysis of matrix-dependent cell migration with a barrier migration assay. Science signaling.

[15] Block ER, Matela AR, SundarRaj N, Iszkula ER, Klarlund JK (2004). Wounding Induces Motility in Sheets of Corneal Epithelial Cells through Loss of Spatial Constraints. Journal of Biological Chemistry.

[16] Nikolić DL, Boettiger AN, Bar-Sagi D, Carbeck JD, Shvartsman SY (2006). Role of boundary conditions in an experimental model of epithelial wound healing. American Journal of Physiology - Cell Physiology.

[17] van Horssen R, Galjart N, Rens JAP, Eggermont AMM, ten Hagen TLM (2006). Differential effects of matrix and growth factors on endothelial and fibroblast motility: Application of a modified cell migration assay. Journal of Cellular Biochemistry.

[18] Koller MR (2004). High-throughput laser-mediated *in situ* cell purification with high purity and yield. Cytometry A.

[19] Zordan MD, Mill CP, Riese DJ, Leary JF (2011). A high throughput, interactive imaging, bright-field wound healing assay. Cytometry A.

[20] Keese CR, Wegener J, Walker SR, Giaever I (2004). Electrical wound-healing assay for cells *in vitro*. Proceedings of the National Academy of Sciences of the United States of America.

[21] Wu SY, Sun YS, Cheng KC, Lo KY (2017). A Wound-Healing Assay Based on Ultraviolet Light Ablation. SLAS technology.

[22] Lo KY, Zhu Y, Tsai HF, Sun YS (2013). Effects of shear stresses and antioxidant concentrations on the production of reactive oxygen species in lung cancer cells. Biomicrofluidics.

[23] Nie FQ (2007). On-chip cell migration assay using microfluidic channels. Biomaterials.

[24] Wei Y (2015). A Tubing-Free Microfluidic Wound Healing Assay Enabling the Quantification of Vascular Smooth Muscle Cell. Migration. Scientific reports.

[25] van der Meer AD, Vermeul K, Poot AA, Feijen J, Vermes I (2010). A microfluidic wound-healing assay for quantifying endothelial cell migration. American journal of physiology. Heart and circulatory physiology.

[26] Felder M (2012). Microfluidic wound-healing assay to assess the regenerative effect of HGF on wounded alveolar epithelium. Lab Chip.

[27] Felder M, Stucki AO, Stucki JD, Geiser T, Guenat OT (2014). The potential of microfluidic lung epithelial wounding: towards *in vivo*-like alveolar microinjuries. Integrative biology: quantitative biosciences from nano to macro.

[28] Wang YC (2019). Wound-on-a-chip: High-throughput 3D wound healing assay with a novel SU-8 mesh chip. Sensor Actuat B-Chem.

[29] Chisti Y (2001). Hydrodynamic damage to animal cells. Critical reviews in biotechnology.

[30] Davies PF, Remuzzi A, Gordon EJ, Dewey CF, Gimbrone MA (1986). Turbulent fluid shear stress induces vascular endothelial cell turnover *in vitro*. Proceedings of the National Academy of Sciences of the United States of America.

[31] Zoro BJH, Owen S, Drake RAL, Hoare M (2008). The impact of process stress on suspended anchorage-dependent mammalian cells as an indicator of likely challenges for regenerative medicines. Biotechnol Bioeng.

[32] Ishibazawa A (2011). Effects of shear stress on the gene expressions of endothelial nitric oxide synthase, endothelin-1, and thrombomodulin in human retinal microvascular endothelial cells. Investigative ophthalmology & visual science.

[33] Ashpole NE, Overby DR, Ethier CR, Stamer WD (2014). Shear Stress-Triggered Nitric Oxide Release From Schlemm’s Canal Cells. Investigative ophthalmology & visual science.

[34] Yamamoto Y, Uno T, Joko T, Shiraishi A, Ohashi Y (2010). Effect of anterior chamber depth on shear stress exerted on corneal endothelial cells by altered aqueous flow after laser iridotomy. Investigative ophthalmology & visual science.

[35] Ramsay MM, Walker LN, Bowyer DE (1982). Narrow superficial injury to rabbit aortic endothelium. The healing process as observed by scanning electron microscopy. Atherosclerosis.

[36] Albuquerque ML, Waters CM, Savla U, Schnaper HW, Flozak AS (2000). Shear stress enhances human endothelial cell wound closure *in vitro*. American journal of physiology. Heart and circulatory physiology.

[37] Gojova A, Barakat AI (2005). Vascular endothelial wound closure under shear stress: role of membrane fluidity and flow-sensitive ion channels. Journal of applied physiology.

[38] Utsunomiya T (2016). Transforming Growth Factor-beta Signaling Cascade Induced by Mechanical Stimulation of Fluid Shear Stress in Cultured Corneal Epithelial Cells. Investigative ophthalmology & visual science.

[39] Molladavoodi S, Robichaud M, Wulff D, Gorbet M (2017). Corneal epithelial cells exposed to shear stress show altered cytoskeleton and migratory behaviour. PloS one.

[40] Yin J, Lu J, Yu FS (2008). Role of small GTPase Rho in regulating corneal epithelial wound healing. Investigative ophthalmology & visual science.

[41] Yin J, Yu FS (2008). Rho kinases regulate corneal epithelial wound healing. American journal of physiology. Cell physiology.

[42] Garanich JS, Mathura RA, Shi ZD, Tarbell JM (2007). Effects of fluid shear stress on adventitial fibroblast migration: implications for flow-mediated mechanisms of arterialization and intimal hyperplasia. American journal of physiology. Heart and circulatory physiology.

[43] Steward RL, Cheng CM, Ye JD, Bellin RM, LeDuc PR (2011). Mechanical stretch and shear flow induced reorganization and recruitment of fibronectin in fibroblasts. Scientific reports.

[44] Gorin DR, Cordts PR, LaMorte WW, Manzoian JO (1996). The influence of wound geometry on the measurement of wound healing rates in clinical trials. Journal of vascular surgery.

[45] Grasso S, Hernandez JA, Chifflet S (2007). Roles of wound geometry, wound size, and extracellular matrix in the healing response of bovine corneal endothelial cells in culture. American journal of physiology. Cell physiology.

[46] Jin W, Lo KY, Chou SE, Mccue SW, Simpson MJ (2018). The role of initial geometry in experimental models of wound closing. Chem Eng Sci.

[47] Salas C (2008). Trypanosoma cruzi: Activities of lapachol and alpha- and beta-lapachone derivatives against epimastigote and trypomastigote forms. Bioorgan Med Chem.

[48] Kung HN (2007). Involvement of NO/cGMP signaling in the apoptotic and anti-angiogenic effects of beta-lapachone on endothelial cells *in vitro*. J Cell Physiol.

[49] Fu SC, Chau YP, Lu KS, Kung H (2011). N. beta-lapachone accelerates the recovery of burn-wound skin. Histology and histopathology.

[50] Kung HN, Yang MJ, Chang CF, Chau YP, Lu KS (2008). *In vitro* and *in vivo* wound healing-promoting activities of beta-lapachone. American journal of physiology. Cell physiology.

[51] Sun YS, Peng SW, Cheng JY (2012). *In vitro* electrical-stimulated wound-healing chip for studying electric field-assisted wound-healing process. Biomicrofluidics.

[52] Huang CW, Cheng JY, Yen MH, Young TH (2009). Electrotaxis of lung cancer cells in a multiple-electric-field chip. Biosensors & bioelectronics.

[53] Varma S, Voldman J (2015). A cell-based sensor of fluid shear stress for microfluidics. Lab Chip.

[54] Lin Jin-Young, Lo Kai-Yin, Sun Yung-Shin (2019). Effects of Substrate-Coating Materials on the Wound-Healing Process. Materials.

[55] Huang, C. H., Hou, H. S., Lo, K. Y., Cheng, J. Y. & Sun, Y. S. Use microfluidic chips to study the effects of ultraviolet lights on human fibroblasts. *Microfluid Nanofluid* **21**, ARTN7910.1007/s10404-017-1922-7 (2017).

