# Supplementary material for: A microfluidics-based wound-healing assay for studying the effects of shear stresses, wound widths, and chemicals on the wound-healing process
Source: Sci Rep. 2019 Dec 27;9:20016. doi: 10.1038/s41598-019-56753-9 (PMC6934480; doi:10.1038/s41598-019-56753-9)
Supplement: Supplementary file 1 — Supplementary Figure. [file 41598_2019_56753_MOESM1_ESM.docx]

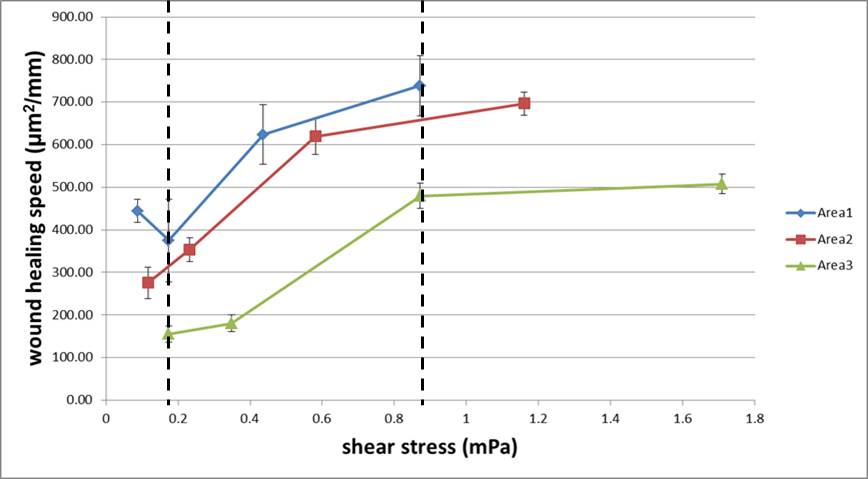


**Figure S1.** Wound-healing speeds under different wound widths and shear stresses.


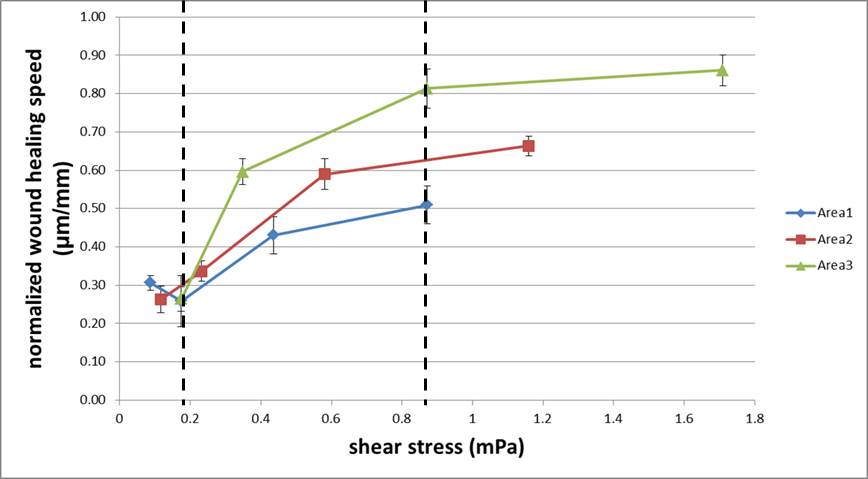


**Figure S2.** Normalized wound-healing speeds under different wound widths and shear stresses.
